# De Novo Radicular Arteriovenous Fistula After Treatment of Spinal Arteriovenous Fistula: A Case Report and Literature Review

**DOI:** 10.7759/cureus.43348

**Published:** 2023-08-11

**Authors:** Tatsuki Mochizuki, Bikei Ryu, Shinsuke Sato, Takakazu Kawamata, Yasunari Niimi

**Affiliations:** 1 Department of Neurosurgery, St. Luke’s International Hospital, Tokyo, JPN; 2 Department of Neuroendovascular Therapy, St. Luke’s International Hospital, Tokyo, JPN; 3 Department of Neurosurgery, Tokyo Women's Medical University, Tokyo, JPN

**Keywords:** interventional radioilogy, endovascular embolisation, spinal vascular malformation, cervical cord, spinal arteriovenous fistula

## Abstract

De novo spinal dural arteriovenous fistulas (AVFs) have been reported as metachronous AVFs However, metachronous spinal AVFs are extremely rare, and their pathogenesis remains uncertain. We report a case of de novo radicular AVF (RAVF) following treatment for spinal AVF at the craniocervical junction (CCJ). We also reviewed the literature and discussed the pathogenesis of metachronous spinal AVF. A 64-year-old male patient diagnosed with spinal AVF at the CCJ supplied from the right C1 segmental artery was treated with Onyx-18 (eV3 Inc, CA, USA) trans-arterial embolization, resulting in partial occlusion. Angiography showed a slight residual shunt two weeks after the embolization without another shunt lesion. A five-year follow-up spinal angiography showed de novo RAVF at the C4 level and complete occlusion of the first AVF. The second AVF was not treated because it was asymptomatic, and the patient remained asymptomatic. De novo RAVF was found to develop five years after the embolization of a CCJ-spinal AVF in a patient. This is the first case of de novo RAVF post-treatment of a spinal AVF. This case demonstrated that RAVF could develop as an acquired disease.

## Introduction

De novo spinal dural arteriovenous fistulas (AVFs) have been rarely reported as metachronous AVFs without a genetic background [1]. Secondary factors such as previous surgery, trauma, or inflammation have been found in the literature to contribute to de novo spinal dural AVF formation [2-9]. Therefore, spinal dural AVFs are accepted as an acquired disease, not congenital [10]. Recently, different from spinal dural AVF, which forms a shunt on the spinal dura mater, radicular AVF (RAVF), which forms a shunt on the nerve root, has been reported in advances of imaging analysis [11]. However, there are no reports of angiographic demonstration of de novo spinal RAVF. Thus, there is still no evidence that points to whether RAVFs are formed secondary to some other factor or are congenital. The pathogenesis of RAVFs is not yet fully elucidated, and the mechanism of their formation is not well understood. Herein, we report a case of de novo cervical RAVF after treatment of a spinal AVF at the craniocervical junction (CCJ). This case demonstrated that RAVF could develop as an acquired disease. We also reviewed the literature and discussed the pathogenesis of de novo spinal AVF.

## Case presentation

History and initial treatment

**Figure 1:**
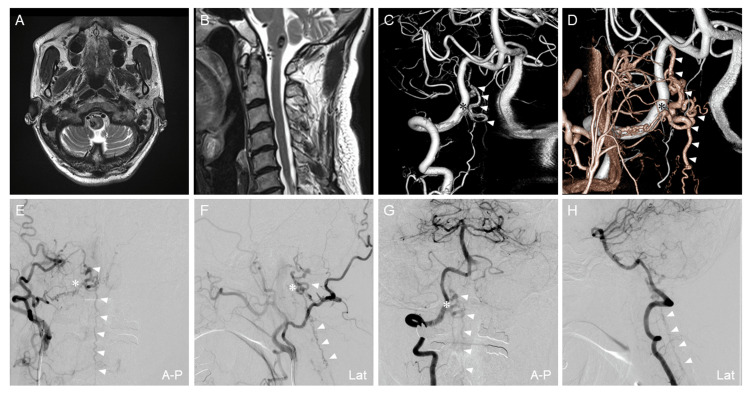
Clinical imaging of CCJ-AVF (first lesion) (A, B) Axial view and sagittal view of brain T2-weighted image of magnetic resonance image show dilated varix at the right medulla oblongata and abnormal flow void in the ventral medulla oblongata. (C, D) 3D-VR image reconstructed from 3D-RA of the right VAG (white vessels) and ECAG (red vessels) shows the structure of AVF. (E, F) Right ECAG shows AVF fed by the muscular branch of the right occipital artery and drained to the ASV. (G, H) Right VAG shows AVF fed by the C1 segmental artery and drained to the ASV. White arrowheads indicate the draining vein. Asterisks indicate the shunt point. 3D-RA, three-dimensional rotational angiography; 3D-VR, three-dimensional volume rendering; A-P, anterior-posterior view; ASV, anterior spinal vein; AVF, arteriovenous fistula; ECAG, external carotid artery angiography; Lat, lateral view; VAG, vertebral artery angiography

A 64-year-old male patient had visited another hospital complaining of right trigeminal neuralgia and gait disturbance for two weeks. Brain MRI showed an abnormal flow void at the anterior surface of the medulla oblongata and varix with perifocal edema at the right medulla oblongata (Figure 1A, 1B). Cerebral angiography showed CCJ-AVF at the C1 vertebral level supplied by the C1 segmental artery originating from the muscular branch of the occipital artery and the right vertebral artery (VA), which drained into the anterior spinal vein (ASV) associated with varix (Figure 1C-1H). Markedly dilated ascending and descending drainers associated with varix were identified at the anterior surface of the spinal cord; no other spinal AVF was identified.

**Figure 2:**
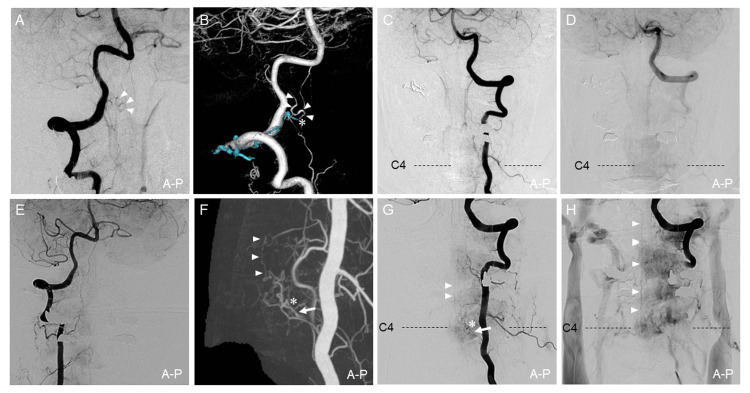
Postoperative follow-up angiography Angiography was performed two weeks after the embolization (A-D). The right VAG (A) and 3D-VR image (B) show a slight residual shunt (blue cast: injected Onyx18). (C, D) The left VAG shows no new lesion or recurrence. Angiography was performed five years after the embolization (E-H). (E) The right VAG shows complete obliteration of the treated first AVF. Maximum intensity projection image (F) of the left VAG and the left VAG (G, H) show de novo RAVF draining into the ASV at the C4 level. White arrowheads indicate draining vessels. Asterisk indicates the shunt point. 3D-RA, three-dimensional rotational angiography; 3D-VR, three-dimensional volume rendering; A-P, anterior-posterior view; ASV, anterior spinal vein; AVF, arteriovenous fistula; VAG, vertebral angiography

Trans-arterial embolization with Onyx-18 (eV3 Inc, CA, USA) was performed two months after onset, resulting in partial shunt occlusion. Transient ischemic symptoms occurred postoperatively because of thrombosis of the varix and venous infarction of the right middle cerebellar peduncle. The right VA angiography performed two weeks postoperatively showed a slight residual shunt (Figure 2A, 2B), and no other shunt lesions were noted (Figure 2C, 2D). Administration of warfarin 3 mg was started after angiography and continued for six months (target prothrombin time-international normalized ratio 2.0-3.0) to prevent progressive venous thrombosis. The patient had an uneventful recovery without worsening the symptoms.

Follow-up

**Figure 3:**
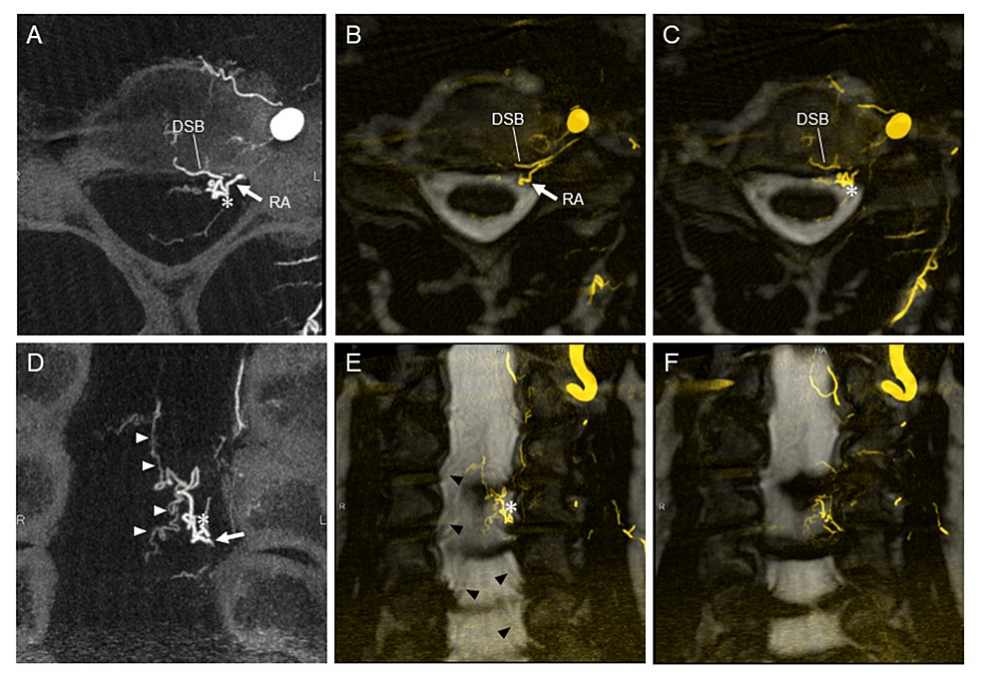
Clinical imaging of de novo RAVF (second lesion) Detailed image analysis shows the de novo RAVF supplied from the radicular artery draining into the ASV (A-F). The shunt is located intradural and on the nerve root. Axial view: (A) 3D-RA MIP image reconstructed from the left VAG, (B, C) MRI fusion images with 3D-RA MIP of the left VAG. Coronal view: (D) 3D-RA MIP image reconstructed from the left VAG, (E, F) MRI fusion images with 3D-RA MIP of the left VAG. White arrowheads show draining veins, black arrowheads show the spinal nerve roots, and an asterisk indicates the shunt point. 3D-RA, three-dimensional rotational angiography; ASV, anterior spinal vein. AVF, arteriovenous fistula; DSB, dorsal somatic branch; MIP, maximum intensity projection; MRI, magnetic resonance image; RA, radicular artery; VAG vertebral artery angiography

MRI showed no sign of recurrence of the treated shunt lesion for the follow-up period of four years. However, the MRI performed five years after treatment showed abnormal vessels on the anterior surface of the cervical spinal cord. The subsequent spinal angiography showed complete occlusion of the treated CCJ-AVF five years later (Figure 2E). However, de novo AVF at the C4 vertebral level draining into the ASV was identified as supplied by the C5 segmental artery originating from the left VA (Figure 2F-2H). The drainer of the new shunt was the same as that of the previous AVF (Figure 1E, 2H). Three-dimensional-rotational angiography (3D-RA) of the left VA and fusion images of 3D-RA with highly T2-weighted volumetric MRI identified that the shunt point of the de novo AVF was located on the C5 anterior root at intradural space (Figure 3). Based on these findings, we diagnosed the second AVF as a de novo RAVF. This lesion was conservatively observed because of the patient’s stable mild dizziness and instability.

## Discussion

Herein, we report a de novo RAVF without a hereditary background. It was angiographically demonstrated five years after spinal AVF embolization, away from the first lesion. To the best of our knowledge, there are no previous reports of de novo RAVF proven angiographically, and this case provides new insights into the pathogenesis of the RAVF of an acquired nature.

Reports of multiple spinal AVFs are rare, and most of them were diagnosed synchronously or on the second follow-up examination. Some of these second lesions were missed during the initial angiography, probably because a complete spinal angiography was not performed [12-15]. De novo AVFs are defined as multiple lesions manifesting at different locations and times, which are even rarer than synchronous multiple spinal AVFs [14]. We reviewed eight cases of true de novo spinal AVFs in Table 1 [1,2,10,13,14,16,17]. All initially diagnosed lesions were spinal dural AVFs except for one intradural AVF. Moreover, all second shunt lesions were dural AVFs except for one intradural AVF [14]. The first and second lesions were more than two vertebrae apart in seven cases [1,2,10,16,17]. The initial treatment for cases where the first and second shunts were less than two vertebrae apart was surgery, which may have contributed to the formation of the second shunt. One case of C1 dural AVF was initially treated by surgery, and the second intradural shunt was discovered at the same vertebral level two days after surgery [14]. This case most likely represents the synchronous occurrence of these two lesions. In the rest of the cases, the second lesion was identified more than three months after treating the initial lesion [1,2,10,13,14].

**Table 1:** Literature review of true de novo spinal AVFs without genetic background *: the period from the first AVF treatment to the second AVF identification; AVF, arteriovenous fistula; DSA, digital subtraction angiography; F, female; Lt, left; M, male; N/R, not reported; RAVF radicular arteriovenous fistula

Author/year	Age (years)/sex	1st lesion						2nd lesion						
		Subtype	Side	Level	Treatment	Outcome	Complete DSA	Subtype	Side	Level	Clinical presentation	Period to identify*	Treatment	Outcome
Barnwell et al., 1991 [16]	69/F	Radiculomedullary AVF	Rt	C1-2	Surgery	No improvement	N/R	N/R	Rt	C6-7	Follow-up examination	N/R	Surgery	No improvement
van Dijk et al., 2002 [17]	62/-	Dural AVF	N/R	Th9	N/R	N/R	N/R	Dural AVF	N/R	Th5	N/R	N/R	N/R	N/R
Sugawara et al., 2005 [10]	71/M	Dural AVF	Rt	Th6	Surgery	Improved	Not performed	Dural AVF	Lt	L1-2	Deterioration (dysuria)	14 months	Surgery	No improvement
Rizvi et al., 2006 [2]	50/M	Dural AVF	Rt	Th8	Embolization and surgery	Improved	Performed	Dural AVF	Lt	L1	Deterioration (paraparesis)	2 years	Surgery	Improved
Dargar et al., 2010 [13]	58/M	Dural AVF	Lt	Th12	Surgery	Improved	N/R	Dural AVF	Rt	L1	Deterioration (paraparesis)	2years	Surgery	Improved
Avecillas-Chasín et al., 2015 [14]	51/M	Dural AVF	Rt	C1	Surgery	N/R	Not performed	Intradural AVF	Lt	C1	N/R	2 days	Surgery	N/R
	72/M	Dural AVF	Rt	Th7	Embolization	Partial improved	Not performed	Dural AVF	Rt	Th12	Deterioration (paraparesis)	5 months	Surgery	N/R
Ren et al., 2019 [1]	61/M	Dural AVF	Lt	Th10	Surgery	Improved	Not performed	Dural AVF	Rt	L1	Deterioration (paraparesis)	4 months	Surgery	Improved
Present case 2023	64/M	Dural AVF	Rt	C1	Embolization	Improved	Not performed	RAVF	Lt	C5	Follow-up examination	5 years	No treatment	No deterioration

Previous trauma, spinal surgery, and inflammation have been reported as causes of the formation of a spinal AVF [2-9]. Specifically, spinal dural AVF is considered to occur secondary to thrombosis or outflow restriction and, thus, congestion of the spinal venous system [15,18]. In the experimental research, venous hypertension was proved to be an inducing factor of dural AVF via overexpression of angiogenic factors [3,19]. In our case, the presence of a spinal AVF could promote the development of a secondary fistula because of medullary venous pressure and concomitant venous stagnation and thrombosis in the adjacent veins [2,13,15]. Treatment of initial spinal dural AVF may also induce secondary AVF development by causing venous thrombosis or venous hypertension [1,2]. Additionally, the presence of a single arteriovenous shunt in the spinal venous system could promote the development of a second fistula because of the subsequently increased medullar venous pressure, the concomitant venous stagnation, and thrombosis in the adjacent veins [2,13,15]. The main drainer of the first AVF and second RAVF was the same ASV in our case, which makes it more likely that the first AVF or its treatment induced the second RAVF. However, there is a possibility that the secondarily diagnosed RAVF existed from the beginning but was angiographically masked by the existence of the first AVF with a higher flow to the same draining vein [20]. In our opinion, it is unlikely because the second RAVF was not identified at the follow-up DSA after two weeks of initial treatment. As this is the only case report showing de novo RAVF, it is difficult to prove what specific factors actually cause de novo RAVF in practice. Further studies are needed to elucidate the pathogenesis of the disease.

Regarding diagnosing RAVFs, it is often difficult to precisely identify the location of the shunt in the spinal nerve root by a single diagnostic modality, including CT, MRI, and angiography. Therefore, some previously reported spinal dural AVFs or perimedullary AVFs may be RAVFs, especially at the cervical level. We identified the location of the shunt by creating a fusion image between high-resolution thin-slice MRI and maximum-intensity projection images of rotational spinal angiography, as we reported elsewhere [11].

Delayed clinical deterioration following a short period of clinical improvement has been reported after the treatment of spinal dural AVFs [14]. In such a case, the possibility of metachronous AVF development should be considered as a cause, in addition to the recurrence of the original lesion and progressive venous thrombosis [2].

## Conclusions

This is a single case report, but this first case of de novo RAVF developed after embolization of a spinal AVF indicates that spinal RAVF can be an acquired lesion like a spinal dural AVF. A patient should be followed-up carefully after treatment for spinal AVF, considering the possibility of the development of de novo spinal AVF, including RAVF.
